# Correlation between osteoarthritis and monocyte chemotactic protein-1 expression: a meta-analysis

**DOI:** 10.1186/s13018-020-02045-2

**Published:** 2020-11-10

**Authors:** Feifei Ni, Yanchao Zhang, Xiaoxiao Peng, Jianjun Li

**Affiliations:** 1 Department of Orthopaedics, Shengjing Hospital of China Medical University, Sanhao Street No. 36, Heping District, Shenyang, 110004 Liaoning People’s Republic of China; 2 Department of Orthopedics, Tianjin Baodi Hospital/Baodi Clinical College of Tianjin Medical University, Tianjin, 301800 People’s Republic of China; 3 Daxing Teaching Hospital of Capital Medical University, Beijing, 102600 People’s Republic of China

**Keywords:** Osteoarthritis, Monocyte chemotactic protein-1, Meta-analysis

## Abstract

**Objective:**

We evaluated the association between monocyte chemotactic protein-1 (MCP-1) and osteoarthritis.

**Methods:**

We searched PubMed, Cochrane Library, Embase, Web of Science, China National Knowledge Infrastructure (CNKI), VIP (Chinese database), and Wan Fang (Chinese database) (before May 10, 2020), with no language limitations. STATA version 12.0 and Revman version 5.3 were used for data analysis. The standard mean difference (SMD) and corresponding 95% confidence intervals (95% CIs) were calculated. Nine clinical studies, including 376 patients with osteoarthritis and 306 healthy controls, were evaluated.

**Results:**

The combined SMDs of MCP-1 expression levels suggested that MCP-1 expression was significantly higher in patients with osteoarthritis than healthy controls (SMD = 1.97, 95% CI = 0.66–3.28, *p* = 0.003). Moreover, subgroup analysis implied that osteoarthritis patients from both Asians and mixed populations had higher MCP-1 expression levels than controls, whereas Caucasians did not (*p* > 0.05). Serum MCP-1 levels (SMD = 2.83, 95% CI = 1.07–4.6, *p* < 0.00001) were significantly higher in patients with osteoarthritis than in controls; however, this difference was not significant in synovial fluid and cartilage tissue. Subgroup analysis for ethnicity showed that MCP-1 levels were significantly higher in Chinese, Dutch, and Brazilian patients with osteoarthritis than in control groups, although significant differences were not observed for American and Italian subgroups.

**Conclusions:**

Our meta-analysis demonstrated that MCP-1 expression levels were higher in patients with osteoarthritis than in healthy controls and that MCP-1 may play important roles in the progression of osteoarthritis. Serum MCP-1 levels may serve as a potential biomarker for the diagnosis of osteoarthritis.

## Introduction

Osteoarthritis (OA) is a type of degenerative articular cartilage disease related to inflammation and is characterized by joint stiffness and pain. OA is most prevalent in middle-aged and elderly individuals, and its incidence has increased in recent years. In patients with OA, joint activity is significantly reduced. If not effectively controlled in a timely manner, OA can cause limb dysfunction and affect the patient’s quality of life [1]. The incidence of osteoarthritis varies in different joints; the most common joint to be affected is the knee, followed by the hip; epidemiological studies have found that the global incidence of OA in the knee is 3.8%, whereas that in the hip is 0.85% [2]. In contrast, the incidence of knee OA in China is 8.1%, whereas that in the United States of America (USA) is 12.1%, which is similar to that in Europe; such incidence is much lower (5.5%) in India [3]. There are currently no curative treatments for OA, resulting in heavy economic burden on patients, families, and society. Additionally, the molecular mechanisms of OA are unclear, and new and effective biomarkers are urgently needed to improve methods for the diagnosis and prevention of OA occurrence and progression [4]. Inflammatory mechanisms are important factors promoting the development of OA. In OA, cytokine and chemokine production, the synovium response, cell infiltration, and inflammatory pathway activation affect disease progression and have been observed in animal models. Chemokines are inducible secreted pro-inflammatory cytokines with a relative molecular mass of approximately 8–10 kDa. The main function of chemokines is to stimulate different types of cells, including neutrophils, monocytes, lymphocytes, and fibroblasts, to undergo chemotaxis, thereby mediating cell aggregation and activation at the site of inflammation and facilitating tissue damage and repair. These molecules also play important roles in various biological processes, such as immune surveillance, organ development, angiogenesis, and immune responses [5–7]. Importantly, many experimental studies have demonstrated that chemokines are involved in the pathogenesis of OA and may be new targets for the early intervention and treatment of OA [8–12].

MCP-1 is an important chemokine secreted by synovial fibroblasts in response to stimulation by the inflammatory cytokines interleukin (IL)-1, tumor necrosis factor-α, and interferon-γ [13]. Notably, MCP-1 is expressed in chondrocytes, osteoblasts, synovial cells, and other cells and plays pivotal roles in bone metabolism and OA [14]. MCP-1 has also been shown to be highly expressed in many diseases [15–19], including OA and rheumatoid arthritis [20, 21]. Additionally, MCP-1 attracts monocytes, resulting in accumulation of monocytes and secretion of cell products to facilitate the OA immune response; this leads to clinical symptoms, such as redness, swelling, and pain [22]. Moreover, abnormal expression of MCP-1 can promote the transformation of monocytes into macrophages in the knee joint capsule, stimulate osteoclasts for bone absorption, induce inflammation, and accelerate the development of OA, leading to joint destruction [23, 24]. MCP-1 also activates monocytes and macrophages to release IL-1 and IL-6 and promotes the production of chemokines and pro-inflammatory cytokines through autocrine and paracrine feedback loops [25]. Reducing the levels of MCP-1 in the joint fluid of patients with OA can alleviate damage to articular cartilage, inhibit the transformation and activation of macrophages, and maintain the stability of the local microenvironment of the joint. Thus, these findings suggest that MCP-1 is closely related to OA; however, some other studies have shown that there is no obvious relationship between MCP-1 expression and OA [26–29]. Thus, the role of MCP-1 in OA remains controversial.

Therefore, in this study, we conducted a meta-analysis to assess the relationship between MCP-1 expression and OA.

## Materials and methods

### Literature search

We searched the following electronic databases without any language restrictions: PubMed, Cochrane Library, Embase, Web of Science, Chinese National Knowledge Infrastructure, VIP, and Wan Fang. The search strategy showed high sensitivity using combinations of the following keywords and MeSH terms: “CCL2” or “MCP-1” or “monocyte chemotactic and activating factor” or “monocyte chemoattractant protein-1” and “Osteoarthritis” or “Osteoarthritis, Spine” or “Osteoarthritis, Knee” or “Osteoarthritis, Hip” or “Knee osteoarthritis” or “Spine osteoarthritis” or “Spinal osteoarthritis” or “Lumbar osteoarthritis” or “Coxarthrosis.”

### Selection criteria

The study selection criteria were as follows: (1) only case-control or cross-sectional studies in the population to explore the relationship between MCP-1 and OA were included, (2) patients in the studies must have met the diagnostic criteria for OA, (3) studies provided means and standard deviations or means and standard errors of MCP-1 levels in patients with OA and healthy controls, and (4) studies must have had sufficient and original data. Studies that did not meet the selection criteria were excluded. If one author published different studies on the same topic, the most recently published study or the study with the largest sample size was selected.

### Data extraction

From the selected articles, two researchers (Feifei Ni, Xiaoxiao Peng) independently extracted and recorded the required information. Disagreements over data or included studies were agreed upon through discussion of all items. The recorded information included surnames of initial authors, region, language, publication year, patient age, MCP-1 detection method, source of sample, and MCP-1 levels in cases and controls.

### Quality of the studies

Two observers (Feifei Ni, Xiaoxiao Peng) used the Newcastle-Ottawa Scale (NOS) to assess the quality of the included studies [30]. The NOS consisted of three factors: (1) patient selection, 0–4; (2) comparability of patients, 0–2; and (3) clinical outcomes, 0–3. NOS scores ranged from 0 to 9, and the quality of the included studies was then categorized as low quality (0–6) or high quality (7–9). When there were disagreements or discrepancies with regard to NOS scores between the two researchers, we sought assistance from a third researcher.

### Statistical analysis

The relationship between MCP-1 levels and OA susceptibility was assessed using standardized mean differences (SMDs) and 95% confidence intervals (95% CIs). We used Cochran’s *Q* test (results displaying *p* < 0.05 were considered significant) and *I*^2^ tests to quantify heterogeneity among studies [31]. A random-effects model was used when there was significant heterogeneity (*p* < 0.05 or *I*^2^ > 50%), whereas SMDs were pooled based on a fixed-effects model [32]. When there was significant heterogeneity, subgroup analysis was performed to identify the potential reasons for the differences in MCP-1 levels between patients with OA and controls. In addition, sensitivity analysis was used to assess whether a single study had an impact on the entire assessment, and the impact of publication bias was analyzed using Egger’s test (results displaying *p* < 0.05 were considered significant), which can be used to evaluate asymmetry visible in a funnel plot [33, 34]. The data were analyzed with the software programs Review Manager 5.3 and STATA version 12.0. This meta-analysis was conducted according to PRISMA guidelines [35].

## Results

### Included studies

**Fig. 1:**
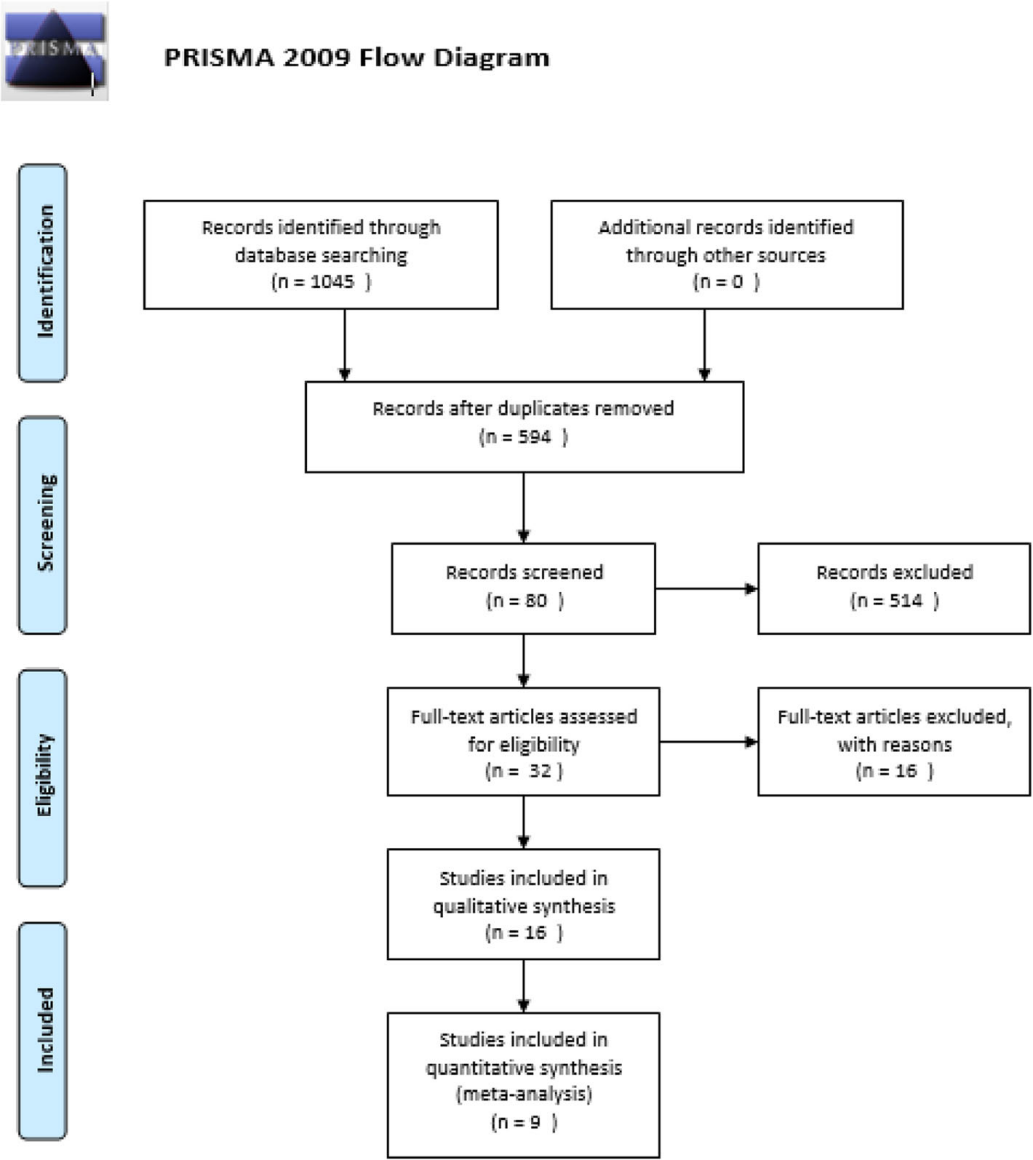
Study selection flow chart

We applied the PRISMA flow diagram to select the studies to be included in our meta-analysis [35]. We selected 1045 potentially relevant articles from eight databases. After deletion of duplicates, there were still 594 studies. By reviewing the titles and abstracts of these studies, we excluded 514 papers because they were obviously irrelevant. The full text of the remaining 80 articles was read, and another 48 studies were excluded (20 studies were not conducted in humans, and 28 studies were not clinical trials), yielding 32 studies. From these 32 studies, 16 studies were excluded (six were not case-control studies, three were not relevant to MCP-1, and seven were not relevant to OA). After removal of these studies, we included nine studies in this meta-analysis [36–44] (Fig. 1).

### Features of this study

**Fig. 2:**
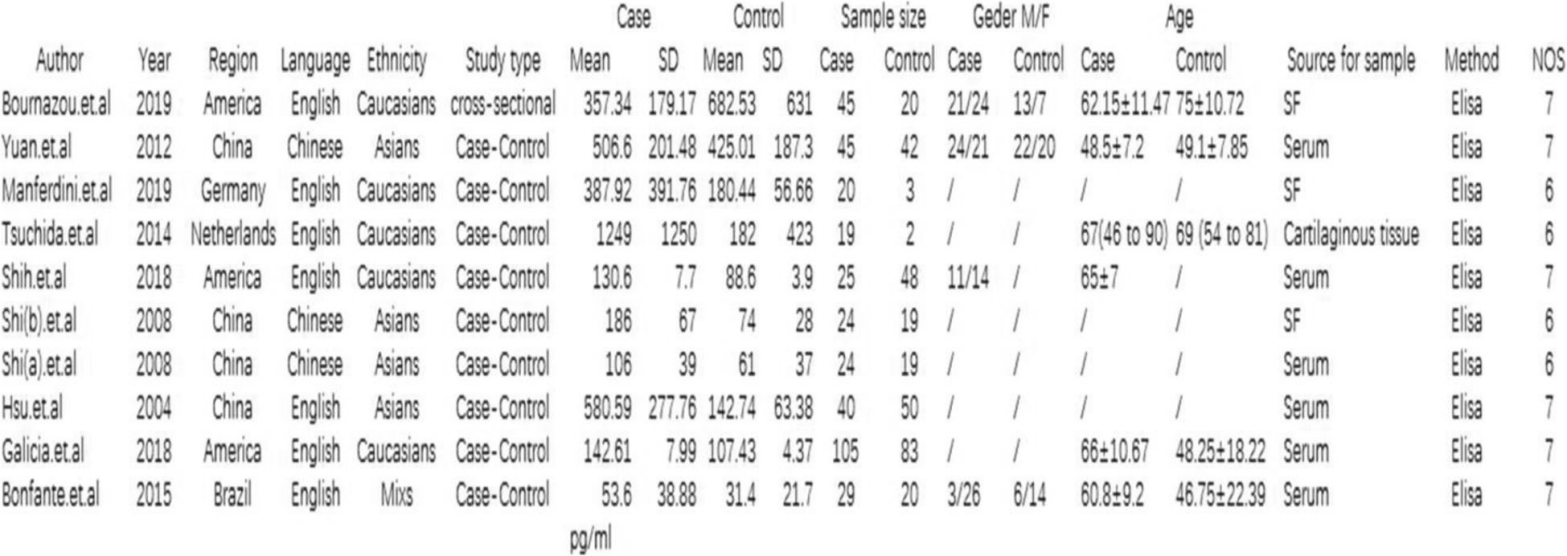
Characteristics of the included studies. M, male; F, female; NOS, Newcastle-Ottawa Scale; SF, synovial fluid; ELISA, enzyme-linked immunosorbent assay

The nine studies included in this study described 376 patients with OA and 306 controls. The basic features of the studies are shown in Fig. 2. The testing method for MCP-1 levels in all nine studies was enzyme-linked immunosorbent assay (ELISA). Methodological quality assessment was performed using NOS, as shown in Fig. 2.

### Meta-analysis of OA

**Fig. 3:**
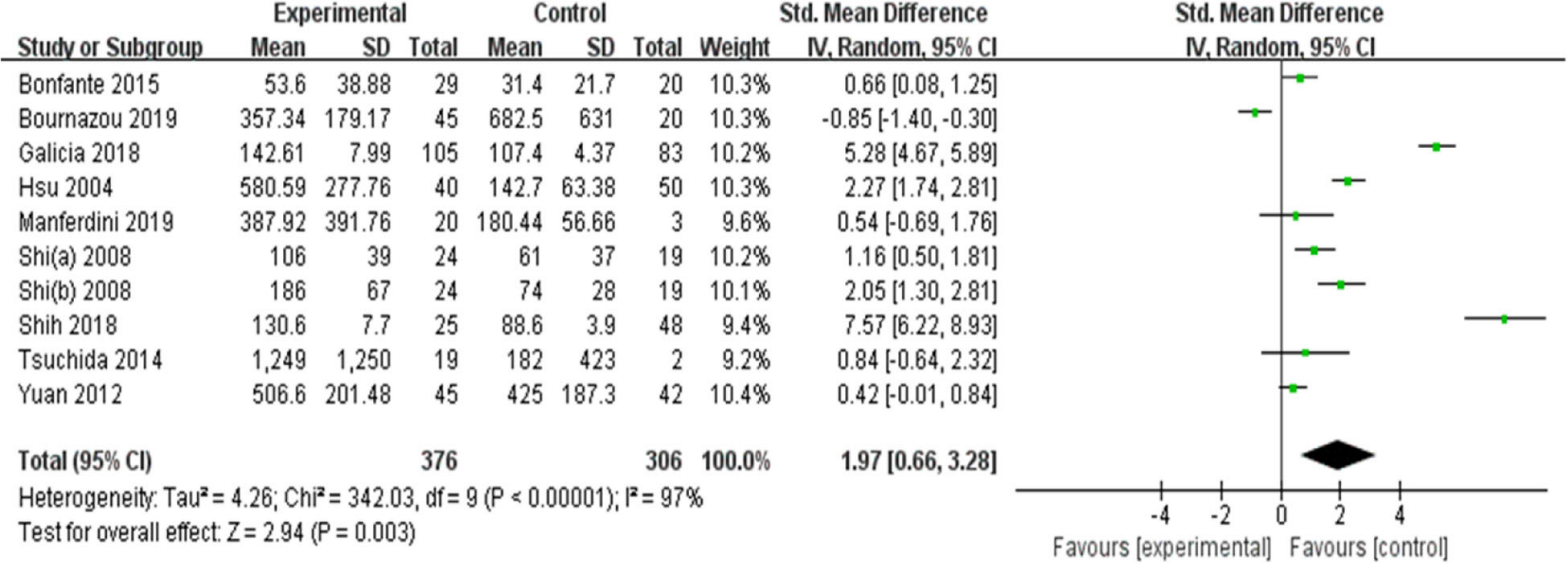
Differences in MCP-1 levels between patients with OA and controls

**Fig. 4:**
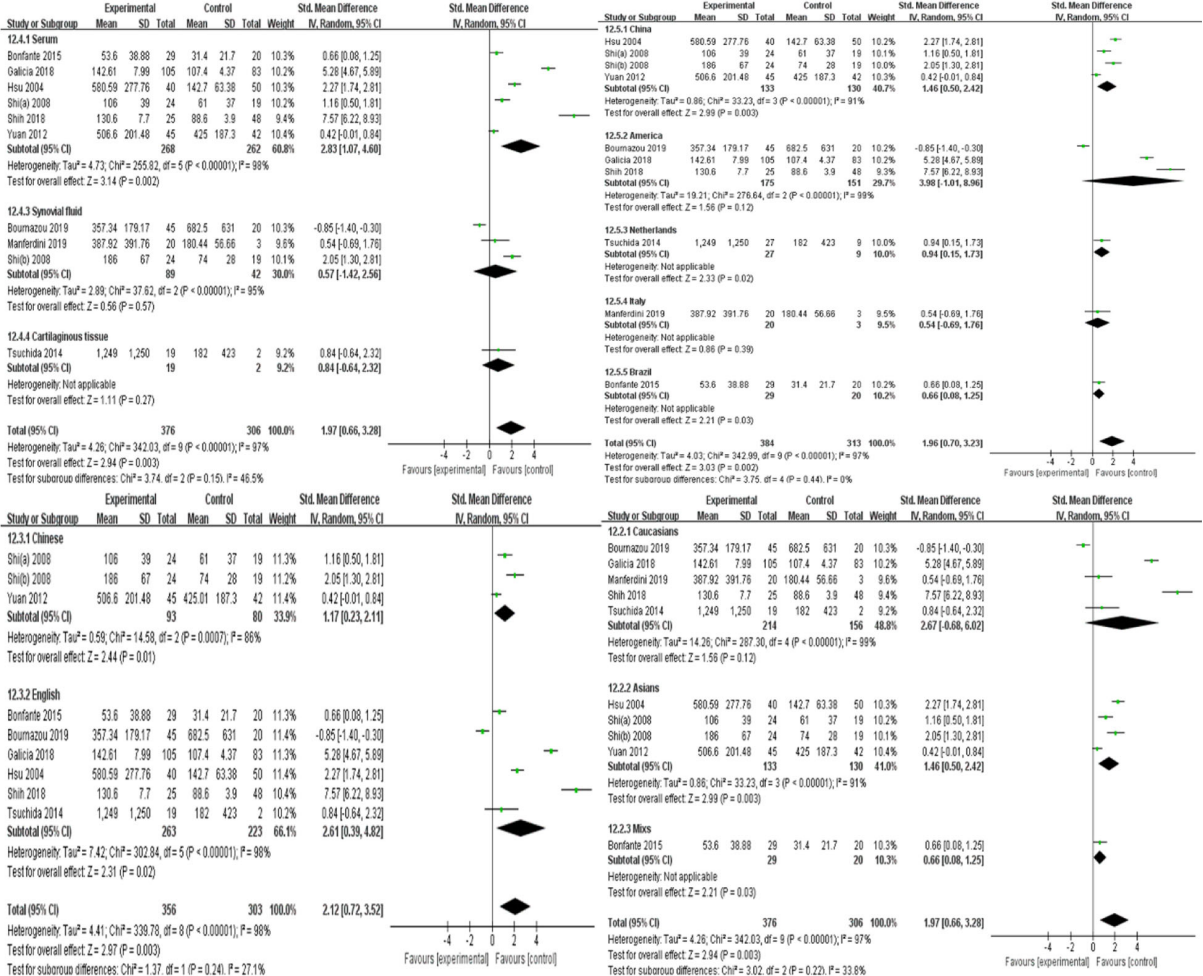
MCP-1 levels according to language, sample source, ethnicity, and country

**Fig. 5:**
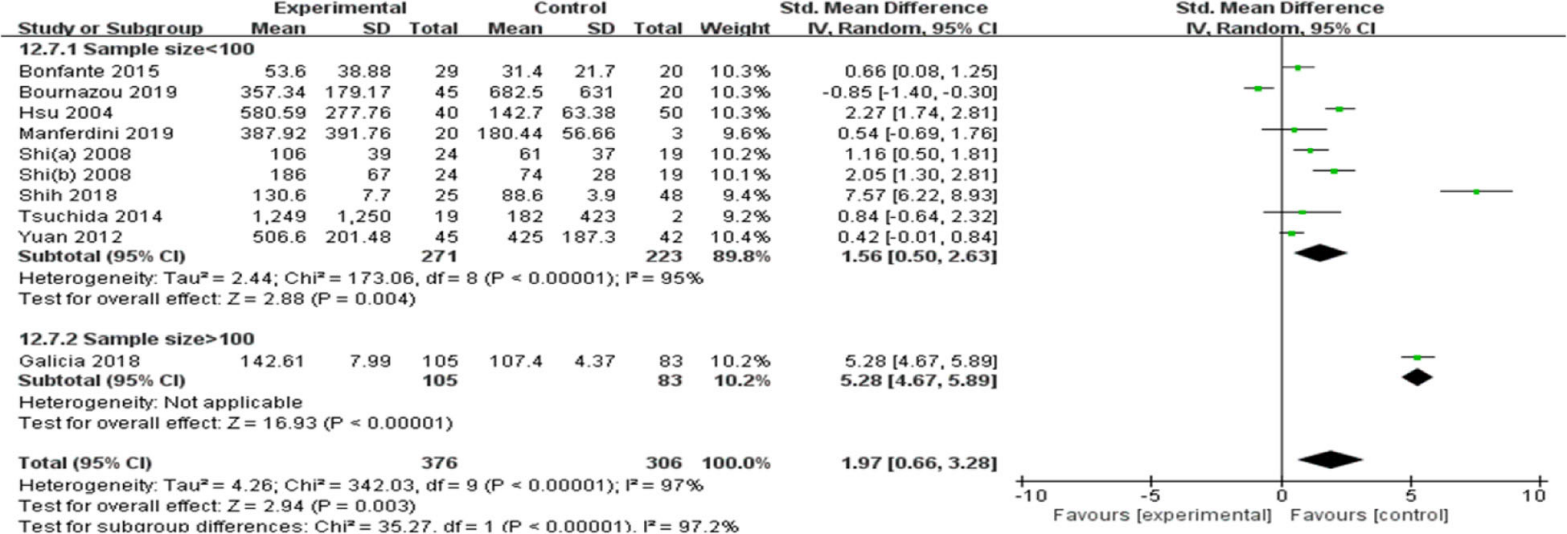
MCP-1 levels according to sample size

Since there was significant heterogeneity among the nine studies (*p* < 0.00001, *I*^2^ = 97%), a random-effects model was used. The results showed that MCP-1 levels were significantly higher in patients with OA than in controls (SMD = 1.97, 95% CI = 0.66–3.28, *p* = 0.003; Fig. 3). In particular, subgroup analysis showed that in both Asians and mixed populations, OA patients had higher MCP-1 serum levels than controls, whereas this result was not observed among Caucasians (*p* > 0.05). Moreover, MCP-1 serum levels in patients with OA were significantly higher than those in the control group, but significant differences were not observed when MCP-1 levels were evaluated in the synovial fluid and cartilage tissue. Further subgroup analysis showed that MCP-1 levels were significantly higher in Chinese, Dutch, and Brazilian OA patients than in the respective controls; however, such differences were not observed in the American and Italian subgroups. Language of the study, sample size, and sample origin were not sources of heterogeneity because heterogeneity was still high after subgroup analysis (Figs. 4 and 5).

### Sensitivity analysis and publication bias

**Fig. 6:**
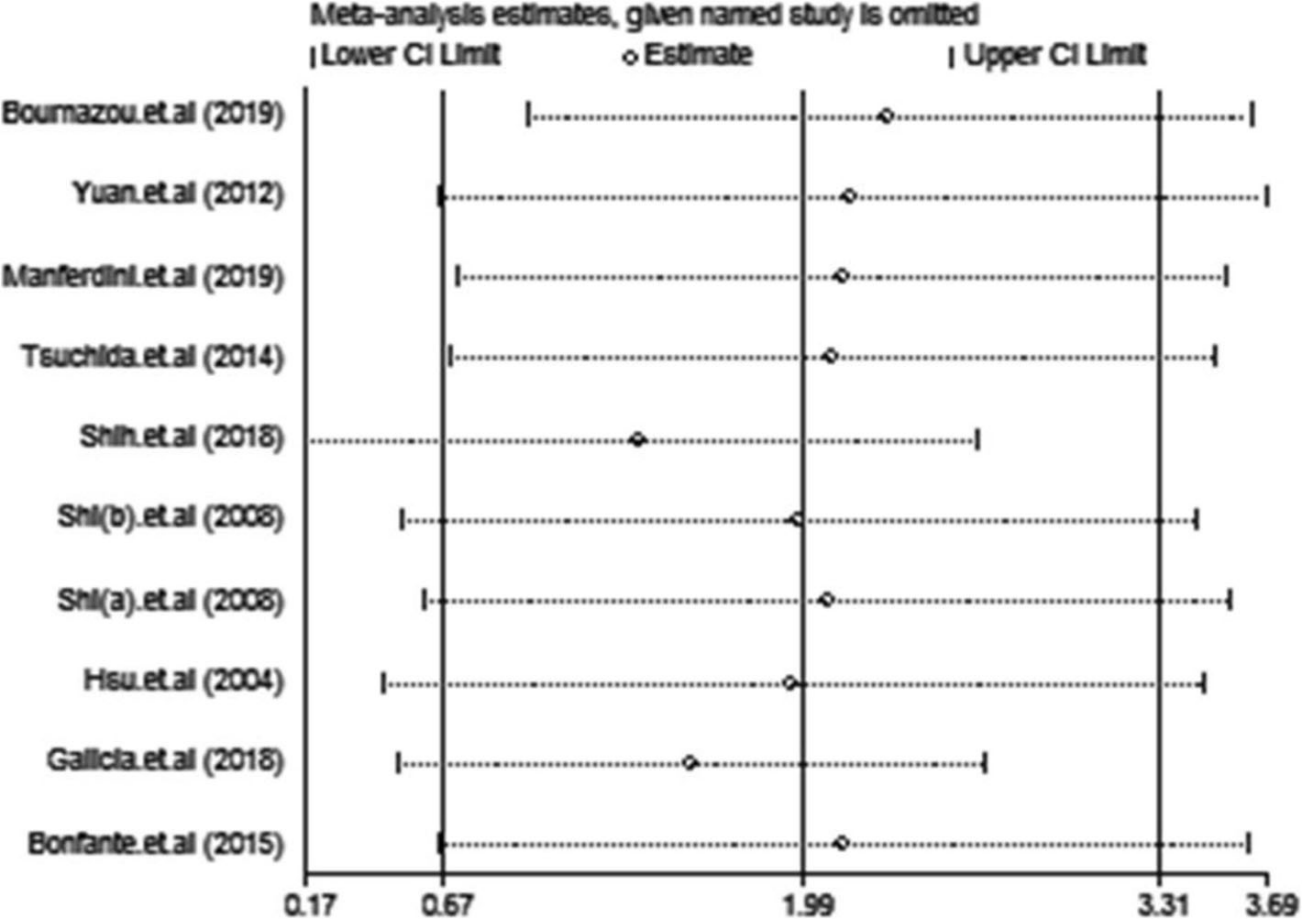
Sensitivity analysis

**Fig. 7:**
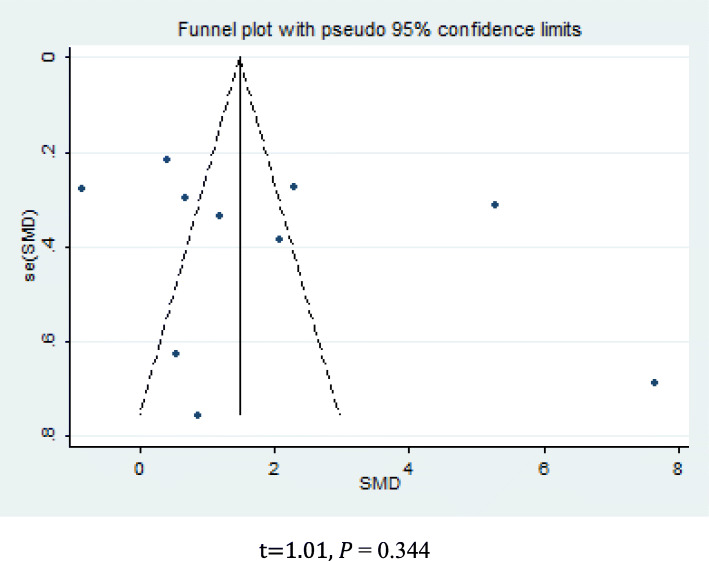
Funnel plots. *t* = 1.01, *p* = 0.344

The results of the sensitivity analysis indicated that none of the studies had an impact on the overall estimate of the association between MCP-1 levels and OA risk. Thus, our meta-analysis data were relatively stable and reliable (Fig. 6). Funnel plots of the nine included studies showed symmetry, and Egger’s tests showed no publication bias (*p* = 0.344; Fig. 7).

## Discussion

In this study, we assessed the relationship between MCP-1 expression and OA. Our meta-analysis showed that MCP-1 expression levels were significantly higher in patients with OA than in controls, indicating that MCP-1 was strongly correlated with the progression of OA. However, the specific mechanisms through which MCP-1 influences OA remain unclear [45]. MCP-1 modulates chemokines expressed by monocytes and is one of the most important chemokines studied to date. Notably, binding of MCP-1 to receptors on the surface of OA chondrocytes can induce the production of matrix metalloproteinases (MMPs) and damage articular cartilage. Additionally, MCP-1 and RANTES promote cartilage catabolism, induce nitric oxide synthase, increase MMP3 expression, and inhibit proteoglycan synthesis [8, 46]. MCP-1 chemotactic monocytes produce large numbers of pro-inflammatory cytokines in OA and promote the progression of OA [47]. Additionally, MCP-1 promotes the transformation of monocyte into macrophages and then to osteoclasts and can induce inflammation downstream [24, 48]. In previous studies, MCP-1 levels in serum and synovial fluid of patients with rheumatoid arthritis were found to be significantly increased compared with that in healthy controls, and high levels of MCP-1 concentrated mononuclear cells from the blood to the articular cavity and then macrophages, activating downstream inflammatory responses and resulting in inflammatory injury [49–51]. Conversely, in a ccl2 knockout mouse model of OA, macrophages were found to accumulate less in OA tissues, and synovitis and cartilage damage were significantly reduced [14]. Interestingly, injection of MCP-1 into the knee joints of mice induced cartilage degradation, and in human studies, chondrocytes and synovial fibroblasts from patients with OA were found to express high levels of MCP-1. MCP-1 has also been shown to reflect the activity of macrophages and to be related to radiological findings and symptomatic inflammation in OA. Thus, MCP-1 may be a reliable indicator of disease severity [52–54]. Taken together, these findings demonstrated that MCP-1 plays important roles in the progression of OA and may contribute to the diagnosis of OA, consistent with our results. Furthermore, MCP-1 levels are directly related to the grade of OA, as more severe OA is associated with higher MCP-1 expression [55]. Therefore, MCP-1 may be applied as a potential biomarker to assess the severity of OA and as a target for clinical treatment.

To further explore the relationship between MCP-1 expression and OA progression and to examine possible sources of heterogeneity, we conducted a subgroup analysis based on language, ethnicity, country, and sample source. Ethnic subgroup analysis showed that MCP-1 serum levels were significantly higher in OA patients than in controls in Asian and mixed populations, but not in Caucasians. One possible explanation could be explained by the different physical qualities, lifestyles, eating habits, genetics, and environments of people belonging to these three ethnic groups. Moreover, country subgroup analysis showed significant differences in all subgroups except the USA and Italy. The results of the Italian subgroup could be explained by the fact that there were only three patients in the control group. Although the results for the two subgroups were not statistically significant, the data still showed higher MCP-1 expression in patients with OA than in the control group, suggesting a potential source of heterogeneity. In addition, a subgroup analysis based on language of the study and sample size showed that these were not sources of heterogeneity; nevertheless, future research should adjust these two factors to avoid heterogeneity in analyses. In contrast, sample source subgroup analysis showed that compared with healthy controls, MCP-1 expression was significantly increased in patients with OA when measured from serum samples, but not when evaluated using synovial fluid and cartilage tissue samples. This phenomenon may be related to the reduced quantity of samples for synovial fluid and cartilage or to the different characteristics of the control group and the fact that only one study was included in the cartilage tissue subgroup. Additionally, different sample sources may affect the diagnostic performance of the assay. Although studies have shown that the levels of MCP-1 in synovial fluid and joint soft tissues are significantly increased [54, 56], such experiments are challenging due to ethical considerations; that is, it is not necessarily ethical to collect synovial fluid and cartilage tissues from healthy volunteers [57]. Thus, these factors may affect the reliability of the results. Therefore, this subgroup analysis confirmed the effectiveness of serum MCP-1 in the diagnosis of OA; however, further studies are needed to determine the correlation between MCP-1 levels in synovial fluid and cartilage tissue and OA severity.

The current meta-analysis has some limitations. First, the number of studies that were included in the analysis and their sample size were relatively small. Moreover, the lack of detailed data prevented to conduct subgroup analysis for other OA-related factors, such as sex, body mass index, and OA Kellgren and Lawrence grade, and this could affect the reliability of the results. Second, language may cause bias. Although there were no language restrictions when we searched the literature, the meta-analysis only included Chinese and English literature, this may lead to a degree of selective bias [58], but no bias was found when using Egger tests, indicating that the data obtained from the studies included in this meta-analysis are reliable and faithfully represent the reality. Third, the ELISA kits used in the studies are diverse and were purchased from different companies. Therefore, we could not determine the sensitivities of these kits. Finally, articles that provided only medians and ranges or upper and lower quartiles were excluded from the meta-analysis. Although a method for data conversion has been reported by Hozo et al. [59], we believe that if we forced the conversion of these data, the conversion results would not be accurate.

Despite the above limitations, this is the first meta-analysis to study the correlation between MCP-1 levels and OA. Thus, we used strict inclusion and exclusion criteria and applied appropriate statistical methods to combine the results of multiple studies in order to achieve strong objectivity, which suggested that our conclusions are reliable and meaningful.

Our results suggested that serum MCP-1 levels were closely related to OA and that MCP-1 played important roles in the pathological progression of OA. At the same time, our results indicated that MCP-1 could be used as a potential biomarker for the diagnosis of OA and also as a therapeutic target for the treatment of OA. Given the limitations of the current study, additional rigorous and detailed experiments with large sample sizes are needed to verify our conclusions.

## Abbreviations

MCP-1: Monocyte chemotactic protein-1
CI: Confidence interval
SMD: Standard mean difference
OA: Osteoarthritis
SF: Synovial fluid
M: Male
F: Female
NOS: Newcastle-Ottawa Scale
PRISMA: Preferred Reporting Items for Systematic Reviews and Meta-Analyses
ELISA: Enzyme-linked immunosorbent assay
